# Novel Smartphone Interventions Improve Cognitive Flexibility and Obsessive-Compulsive Disorder Symptoms in Individuals with Contamination Fears

**DOI:** 10.1038/s41598-018-33142-2

**Published:** 2018-10-23

**Authors:** Baland Jalal, Annette Brühl, Claire O’Callaghan, Thomas Piercy, Rudolf N. Cardinal, Vilayanur S. Ramachandran, Barbara J. Sahakian

**Affiliations:** 1 University of Cambridge School of Clinical Medicine, Department of Psychiatry and Behavioural and Clinical Neuroscience Institute, Herchel Smith Building for Brain and Mind Sciences, Cambridge, CB2 0QQ Cambridge Biomedical Campus, UK; 2 Liaison Psychiatry Service, Cambridgeshire & Peterborough NHS Foundation Trust/Cambridge University Hospitals NHS Foundation Trust, Cambridge Biomedical Campus, Cambridge, CB2 0QQ UK; 3 University of California at San Diego, Center for Brain and Cognition, La Jolla, USA; 4 Department of Psychiatry, Psychotherapy and Psychosomatics, Psychiatric Hospital, University of Zurich,, Zurich, Switzerland

## Abstract

One type of obsessive–compulsive disorder (OCD) is characterized by contamination fears and compulsive cleansing. Few effective treatments are available for this debilitating condition. Compulsive symptoms, such as excessive washing, are believed to be mediated by cognitive inflexibility—arguably the most striking cognitive impairment in OCD. In this study, we investigated the effects of two novel smartphone interventions on cognitive flexibility and OCD symptoms in healthy individuals with OCD-like contamination fears. In the first intervention, participants watched a brief video recording of themselves engaging in handwashing on a smartphone, four times a day, for a total of one week (*N* = 31). The second intervention was similar except that participants watched themselves repeatedly touching a disgust-inducing object (*N* = 31). In a third (control) “intervention”, participants watched themselves performing sequential hand movements (*N* = 31). As hypothesized, the two smartphone interventions, unlike the control, improved cognitive flexibility; as assessed on the Intradimensional–Extradimensional Set Shifting task (a sensitive marker of cognitive flexibility). The two interventions, unlike the control, also improved OCD symptoms (measured with the Obsessive–Compulsive Inventory–Revised and Yale–Brown Obsessive–Compulsive Scale). Finally, we found high levels of adherence to the interventions. These findings have significant clinical implications for OCD.

## Introduction

Obsessive–compulsive disorder (OCD) can be a highly debilitating condition associated with considerable distress^1^. One of the most common types of OCD, affecting up to 46% of OCD patients, is characterized by severe contamination fears and excessive washing behaviors^2^. These patients feel anxious even after incidents of minor “contamination” (for example, touching a door knob), and may spend up to several hours painstakingly washing and scrubbing their hands, sometimes causing bleeding and skin damage^3^. The first-line non-pharmacological treatment for this type of OCD is a form of cognitive behavioral therapy (CBT) called “exposure and response prevention” (ERP)^4^. During ERP, an individual is progressively exposed to a “contaminated” object (e.g., a toilet seat) to feel the rise of anxiety (exposure) and then abstains from engaging in compulsive behaviors (response prevention); this in turn helps the person experience the decrease of anxiety and overcome their fear, resulting in habituation^1^. While ERP has been the treatment of choice for OCD since the early 1980s, it is not effective in many cases; indeed, a great many do not benefit from or tolerate this therapy^5^. At present, as many as 40% of OCD patients fail to respond to treatment (either by CBT or serotonin reuptake inhibitor drugs)^6^. Developing novel non-pharmacological treatments for OCD therefore represents a critical unmet need.

Innovative technology-based therapies, for example using smartphones^7,8^— “technology-based personalized medicine”—have the potential to transform OCD treatment. By moving therapy out of the clinician’s office and into the hands of the patients themselves, these interventions can be tailored to the specific needs of individual patients, which could ultimately improve treatments. Such interventions, unlike standard CBT and drug treatments, are inexpensive and can facilitate psychotherapy by making it readily available to patients, and by encouraging them to take a more active role in their treatment strategies. Indeed, smartphone interventions are especially well suited to modernized societies where people, more than ever, are becoming reliant on such technology.

Our group recently revealed the impact of vicarious, rather than direct, exposure on OCD-like contamination fears, with potential implications for therapy^9^. Participants with OCD symptoms reported experiencing disgust when watching someone else touching a contaminated object (e.g., fake feces). More intriguingly, after the participants had contaminated themselves, they obtained relief from merely watching someone else washing their own hands. The authors refer to this effect, the induction of emotions and sensations (e.g., disgust and relief) vicariously, as “vicarious exposure”^9^. These findings are consistent with research showing that brain regions involved in the processing of disgust, such as the insula, become activated not only when people experience this emotion themselves^10^, but also when they watch someone else experience disgust^11^. This empathetic response has been found to activate the anterior cingulate cortex^10^, and is thought to be mediated by the activity of the mirror neuron system^11,12^.

If vicarious observation of repetitive behaviors can play a functional role for the patients that is similar to actually performing them, it could be used for developing smartphone interventions for OCD using “vicarious exposure” procedures. It may be that for some OCD patients, merely watching video footage of themselves washing their hands, when feeling contaminated, brings about sufficient relief to eliminate the urge to engage in the actual hand-cleansing behavior. Even if the cleansing urge is only partly eliminated, that would still have a potential therapeutic advantage of reducing high levels of acute stress and anxiety, known to worsen compulsive symptoms. It is conceivable that over time such short-term relief would lead to higher-level cognitive realization that refraining from the compulsion brings no harm, thus decoupling the behavior from the stimulus. Another possible application of this therapeutic procedure is that it could serve as a benign substitute compulsion. In cases of treatment-refractory OCD, for instance, this approach would be an alternative way to prevent skin damage due to excessive handwashing, and, with smartphones readily accessible, might reduce the time spent on performing compulsive behaviors.

Similarly, if contamination sensations can be induced vicariously, smartphone interventions could be aimed at desensitizing OCD patients to stimuli that provoke disgust and anxiety. For example, if OCD patients repeatedly watched video footage of themselves touching disgust-inducing objects, such exposure might eventually lead to habituation—that is, diminished emotional responsiveness to the aversive stimulus. The aim of this intervention would be analogous to ERP, except that it would be inexpensive, and allow patients to complete at least part of their therapy in the absence of the therapist, making it transportable and easily accessible. This type of “vicarious desensitization therapy” is conducted in a real-life setting where patients’ contamination fears and washing compulsions arise in their day-to-day lives, as opposed to the artificial environment of the clinic. This might be contextually more appropriate and accelerate stimulus generalization, potentially increasing the therapeutic efficacy.

Compulsive symptoms such as excessive washing behaviors are believed to be mediated by cognitive inflexibility (impaired “set shifting”). This is perhaps the most striking cognitive/executive impairment in OCD, characterized by the inability to shift attentional focus^13^. Indeed, growing evidence shows that cognitive inflexibility represents a core feature and biomarker of the neurocognitive profile of OCD (for meta-analyses see^14,15^) and a candidate neurocognitive endophenotype^16,17^. Such cognitive flexibility or set shifting deficits in OCD are mediated by abnormal activation of fronto-striatal circuitry (e.g., dorsolateral/ventrolateral prefrontal and striatal regions)^18,19^. Interestingly, fronto-striatal dysfunction in OCD is amenable to treatment^20^. A key measure of cognitive flexibility on which OCD patients consistently perform less well than healthy controls is the Intradimensional–Extradimensional (IED)^21^ Set Shifting task^22^ of the well-validated Cambridge Neuropsychological Test Automated Battery (CANTAB)^23^ (for a review on the IED task in OCD see^24,25^). A series of studies have demonstrated that performance on the crucial extradimensional shift (EDS) stage of the IED task (conceptually similar to the Wisconsin Card Sort Test [WCST]^26^) is impaired in OCD^19,25,27–29^, making it a sensitive marker of cognitive flexibility.

In the current study, we investigated the effects of two novel smartphone interventions on cognitive flexibility and OCD symptoms in healthy individuals with OCD-like contamination fears. The first intervention tested the effect of participants watching a brief video recording of themselves engaging in handwashing, four times a day, for a total of one week (washing condition). The second intervention tested the effect of participants watching a video recording of themselves repeatedly touching a disgust-inducing object, four times a day, for a total of one week (“contamination” condition). A third, control intervention was identical to the two experimental interventions, except that participants instead watched a video recording of themselves performing arbitrary hand movements. Specifically, given the key role of cognitive inflexibility as a mediator of compulsivity in OCD, we hypothesized that following the two active smartphone interventions, participants would improve on the EDS stage of the IED task (a key marker of cognitive flexibility), but not following the control intervention.

## Methods

### Participant selection

Participants were recruited from the local community via online forums, flyers, newspaper adverts, mailing lists, and volunteer databases. Individuals were selected for the study if they endorsed elevated contamination fears, as defined by a score of at least 10 points on the Padua Inventory Contamination Fear Subscale^30^ (PI CF) during the initial telephone screening and at least 9 on the PI CF during the first laboratory testing session. Study participation was restricted to those aged between 18–65, who were proficient in English, and without a history of psychiatric disorders. During the initial phone interview potential participants were screened using the Modified Mini Screen (MMS)^31^. If they endorsed any of the questions on the MMS screen, they were administered the specific diagnostic module of the Mini International Neuropsychiatric Interview (MINI^32^) relevant to their answer. If any of their answers on the MINI indicated a possible clinical diagnosis, the individual was interviewed by an experienced psychiatrist to rule out a clinical diagnosis.

### Procedure

Participants were randomized to one of three conditions: the washing condition (smartphone intervention I), contamination condition (smartphone intervention II), or the control condition. Participants in the three conditions were actively matched for age, sex, years of education and level of contamination fears.

Participants attended two sessions, 8 days apart. This study was approved by the University of Cambridge’s Psychology Research Ethics Committee and all research was performed in accordance with the relevant guidelines and regulations. All participants provided written informed consent prior to participation in the study and received monetary compensation for their travel costs and time. In both sessions, they completed a battery of clinical measures and neuropsychological tests (described in more detail below). At the end of session one, they completed a 30-second video recording that would form the basis of the smartphone intervention.

**Figure 1:**
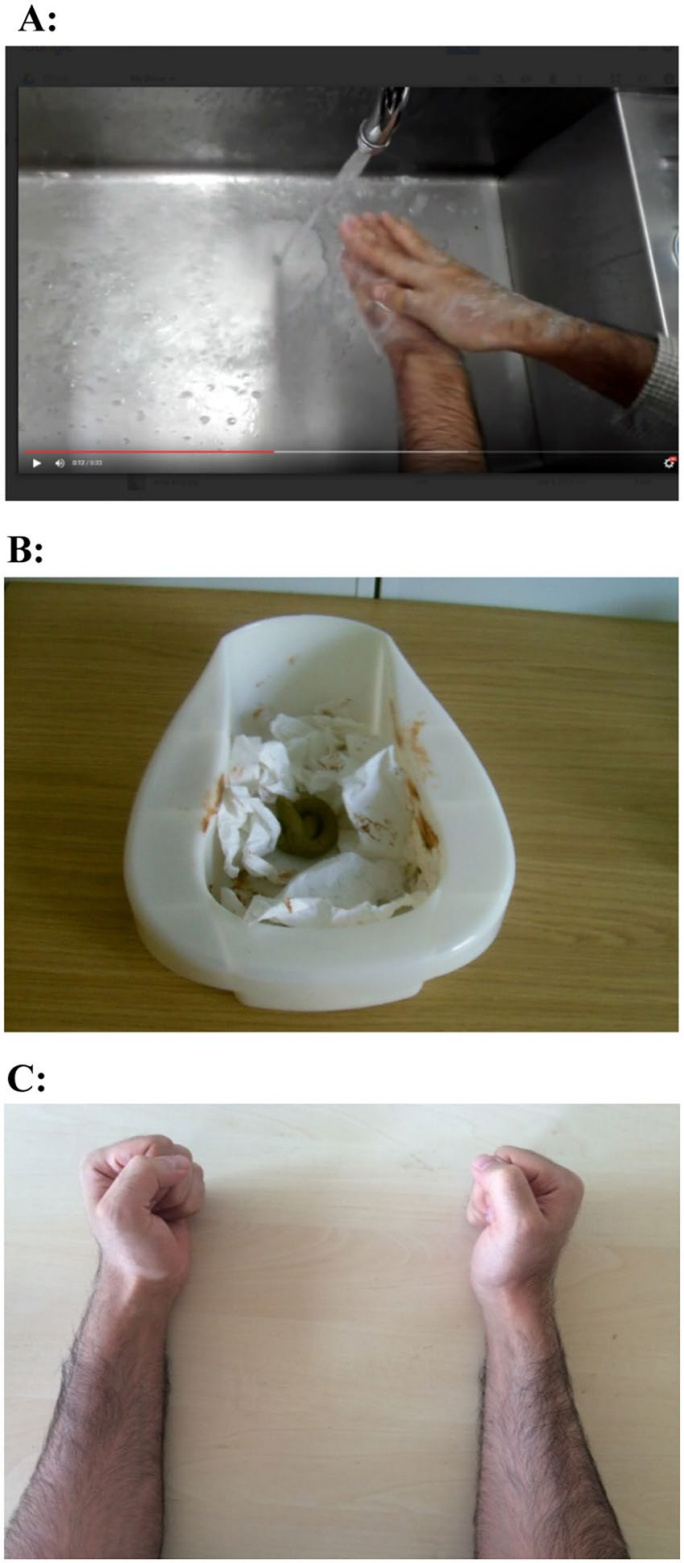
(**A**) The video footage used in the “washing” condition. (**B**) The “disgust stimulus” used for the video footage in the “contamination” condition. (**C**) The hand movements performed for the video footage in the control condition.

Participants in the washing condition were recorded while washing their hands with soap at a basin (Fig. 1A). Those in the contamination condition were recorded while repeatedly touching toilet paper in a bedpan. This toilet paper was stained (using food substances) to resemble feces and placed around a fake feces replica. An unpleasant odor was sprayed on this object to increase its authenticity. Consistent with our previous methodology, participants were not informed that the feces were fake (Fig. 1B)^9,33^. (During piloting we found that participants rated “fake feces” as more disgusting than “fake vomit” and “fake blood”. This result is consistent with our previous research^9^.) Participants in the control condition were shown a sequence of hand movements (a cutting motion on the table, followed by a fist, and then palm down with fingers extended; Luria’s Hand Sequences, i.e., “cut, fist, and slap”^34^) (Fig. 1C). They were filmed while making these movements with both hands resting on a table. All the videos were recorded such that they only showed the participants’ hands and arms, and simulated the vantage point of the participants looking down at themselves.

The experimenter then installed the smartphone application on the participants’ smartphones and uploaded the video recording to the application. The experimenter instructed participants how to use the application, and participants completed a practice trial on their smartphones in the presence of the experimenter to ensure that they had understood the instructions. The first visit was then complete. Thereafter, participants completed the smartphone intervention for seven days, as they went about their daily lives. After a week of using the smartphone application, participants returned for a second visit and debriefing.

### Smartphone application

**Figure 2:**
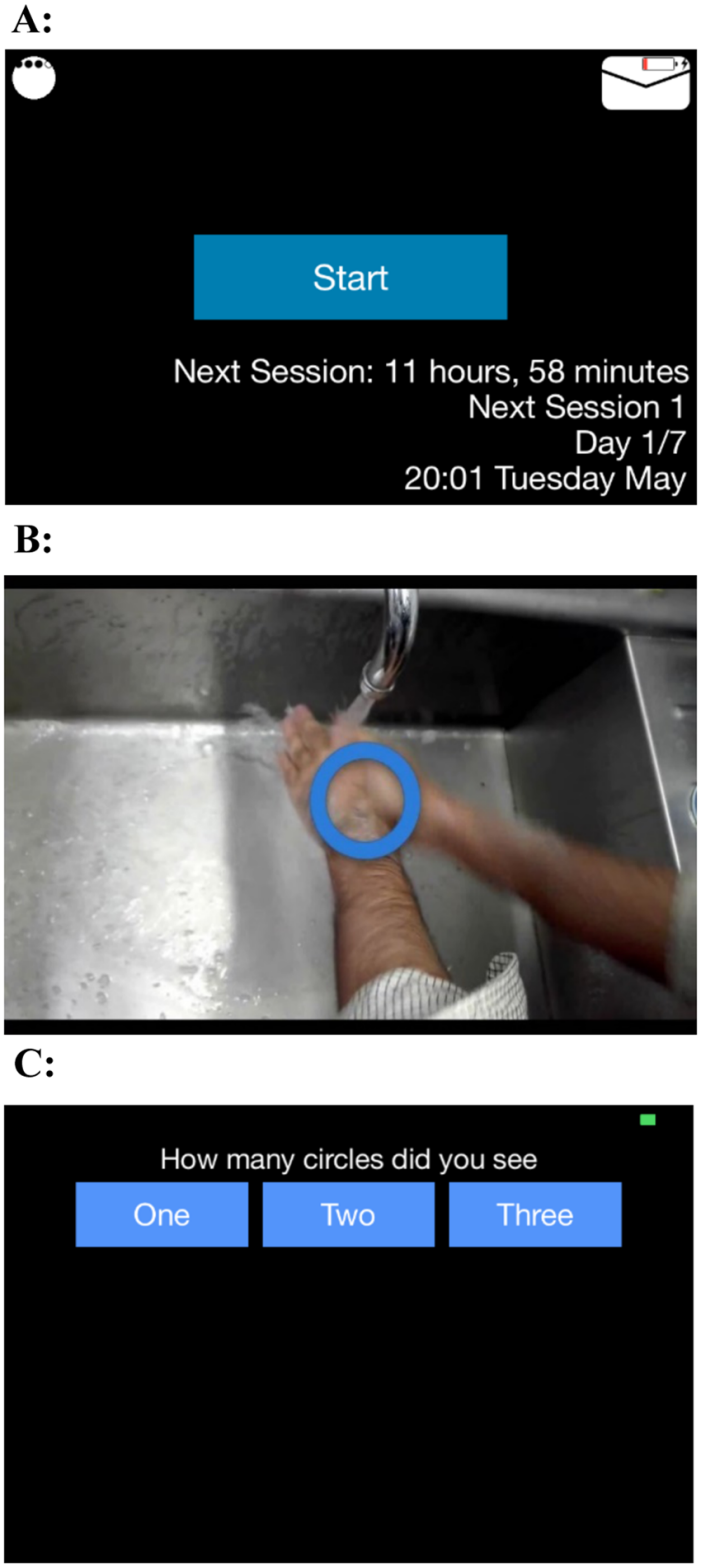
(**A**) The default start screen of the smartphone application. (**B**) The flashing circles superimposed on the video footage to track that participants were watching. (**C**) The screen where participants indicated the number of circles they saw, immediately after the video finished playing.

The smartphone application was designed to be compatible with iPhones (model 4S or newer), iPod Touch devices, and Android-based smartphones. A smartphone (Samsung Galaxy S3) and Apple iPod Touch (6^th^ generation, with a 4-inch diagonal widescreen display), similar in dimensions to many widely used smartphones, were also available for participants to use during the duration of the study to avoid technical obstacles arising from running the application. The primary function of the smartphone application was to play a video recording (30 seconds) of participants either: (1) washing their hands, (2) touching a “contaminated” object, or (3) performing a sequence of arbitrary hand movements. Participants were instructed to use the application four times a day for seven days; i.e., at least once during the following time windows: 8 am to 12 pm; 12 pm to 4 pm; 4 pm to 8 pm; and 8 pm to 12 am. The default screen of the application showed a start tab at the center that participants had to touch to play the video recording; the default screen also showed which session of the day they had to complete (1–4), days remaining of the intervention (1–7), and time remaining before the next session. A virtual envelope was displayed at the top right of the screen, which participants could touch in order to e-mail the data to the experimenter (Fig. 2A). Participants were asked to press the envelope after each session so that the experimenter could track their progress. (As the virtual envelope did not function on all smartphones and iPod devices, participants were asked in these cases to update the experimenter on their progress via e-mail or SMS at least once a day.) To ensure that participants viewed the video at all times, while watching they were randomly presented with either one, two or three flashing circles superimposed on the video recording (approx. 2 seconds per flash) (Fig. 2B). Once the video stopped playing, they were asked to indicate the number of circles they saw (Fig. 2C). When participants had completed the session, the start tab disappeared from the screen and participants could no longer initiate a session; it would reappear once it was time to undertake a session again.

### Contamination fear, OCD symptomatology, and mood assessment

Before and after the intervention, participants completed the following validated self-report questionnaires and clinical interviews to assess factors related to contamination fears, OCD symptomatology, and mood.

Padua Inventory Contamination Fear Subscale (PI CF): the PI CF^30^ is a 10-item scale assessing the presence and severity of contamination fears and washing compulsions. Items are scored on a 5-point Likert scale and scores are generated by adding the item scores; the possible range of scores is 0–40.

The Obsessive–Compulsive Inventory—Revised (OCI-R): the OCI-R^35^ is a self-report scale that assesses distress levels associated with OCD symptoms in the last month. It consists of 18 questions rated on a 5-point Likert scale and scores are generated by adding the item scores; the possible range of scores is 0–72.

Yale–Brown Obsessive–Compulsive Scale (Y-BOCS): the Y-BOCS^36^ is a semi-structured interview that assesses OCD symptom severity (obsessions and compulsions) and response to treatment. Scores are generated from 10 items, each rated on a 5-point Likert scale. The possible range of scores is 0–40. The version of the Y-BOCS employed in the current study ranged from 1–40, with item 10 (measuring “degree of control over compulsive behavior”) rated on a 4-point Likert scale (1–4).

Spielberger State–Trait Anxiety Inventory (STAI-S/T): participants completed the STAI-S/T^37^ comprising 40 items assessing state and trait levels of anxiety. Each subscale on the STAI consists of 20 items that are rated on a 4-point Likert scale. Scores are generated by adding the item scores, with a total score ranging from 20 to 80 on each subscale.

Beck Depression Inventory–II (BDI-II): all participants also completed the BDI-II^38^, a 21-item self-report measure of depression rated on a 4-point Likert scale. Scores are generated by adding the item scores; the possible range of scores is 0–63.

### Cognitive flexibility assessment

Before and after the intervention, the following neuropsychological task of cognitive flexibility was administered from the CANTAB battery (www.cambridgecognition.com)^23^ via a touch-sensitive screen.

Intradimensional–Extradimensional Set Shifting task: the IED^21^ is an attentional set shifting measure^22^. The task starts with the participant seeing two colored geometric shapes. Participants are required to touch the correct shape on the screen and feedback is provided after every response. They can therefore learn which of the two shapes is correct through trial and error. After six consecutive correct responses, the stimuli and/or rules are changed. These shifts are intra-dimensional as the shapes only differ on one dimension (shape). Later, white lines are superimposed on the two shapes and participants learn over the course of several stages that these lines are an irrelevant dimension. During the crucial extradimensional shift (EDS) stage, the white lines become the only relevant dimension. The EDS stage indexes cognitive flexibility; that is, assessing the ability to shift attention away from previously relevant stimulus dimensions to a novel (previously irrelevant) one. A key outcome measure on this task is errors made in the EDS stage. Another outcome measure is pre-extradimensional shift (pre-EDS) errors; that is, errors in the stages before the extradimensional shift.

### Statistical analyses

For the IED task, we analyzed EDS errors as the primary dependent variable of interest. A secondary variable of interest was pre-EDS errors (total task errors, across all stages, minus EDS errors). Other dependent variables of interest included the PI CF, OCI-R, and Y-BOCS.

Two participants in session 1 were missing a single score each on the Y-BOCS and one participant was missing a single Y-BOCS score in session 2. Their scores were rescaled to the maximum possible total (i.e. adjusted score = full scale maximum × subject’s score ÷ subject’s possible maximum).

Dependent variables before and after the intervention were analyzed with an analysis of covariance (ANCOVA). As predictors, we used the subjects’ pre-intervention scores on the same task (baseline performance: a continuous covariate) and the intervention (a factor with 3 levels). An initial analysis was performed in which the covariate × factor interaction was included (a separate-slopes model). If this interaction term was not significant and the interaction model was not superior to a model without the interaction, by a χ^2^ model comparison test, the simpler ANCOVA model without the interaction (a single-slope model) was used. Since subjects were randomized to interventions, with equal group sizes, sequential (type I) sums of squares (SS) were used, prioritizing treatment effects over baseline performance to maximize power. (This method differs from type II/III SS in its treatment of that portion of variance in the dependent variable potentially attributable to either the treatment effect or baseline performance, due to correlation between the two predictors. Given that subjects were randomized to equally sized groups, any such correlation is by definition random; any such variance was attributed to the treatment. This does not alter the attribution of variance attributable to the treatment but not to baseline performance, or that attributable to baseline performance but not the treatment—the latter being an important contributor, as baseline performance strongly predicts subsequent performance^39^). Following a significant main effect of treatment, pairwise comparisons were made with separate ANCOVAs; in this specific case of pairwise comparisons used only following a significant main effect, no further family-wise error rate correction is necessary^40^; however, the sub-ANCOVAs were not constrained to use the slope from the overall ANCOVA.

For all measures, the distribution of residuals was checked with Q–Q plots and the Shapiro–Wilk test. Preliminary examination of untransformed scores showed that for some dependent variables, the residuals deviated substantially from a normal distribution, with positive skew and leptokurtosis (e.g. for EDS errors). Such variables were therefore transformed with a log_10_(*x* + 1) transformation prior to final analysis^39^.

## Results

### Demographics and baseline measures

The initial subject pool consisted of 96 participants. Three participants were subsequently excluded: one participant for missing data (i.e., 50 percent of their smartphone sessions), one participant due to a technical error on the smartphone application, and one participant for failing to attend the final laboratory assessment, due to a scheduling conflict, despite completing the 7-day intervention. The final subject pool thus comprised 93 participants (washing *n* = 31; contamination *n* = 31; control *n* = 31). Sixty participants (64.5 percent) were female and 33 (35.5 percent) were male. The age range was 18–64 years (*μ* = 25.2, *SD* = 8.0). (For demographics and baseline clinical measures, see Table 1.)

**Table 1:** Demographic and clinical characteristics for the final randomized groups (*μ*, mean; *SD*, standard deviation; *n*, sample size; *F*, *F* statistic; *Χ*^2^, chi-square statistic; *df*, degrees of freedom; *p*, *p* value; NS, non-significant; PI CF, Padua Inventory Contamination Fear Subscale; OCI-R, Obsessive–Compulsive Inventory—Revised; Y-BOCS, Yale–Brown Obsessive–Compulsive Scale; STAI-T, Spielberger Trait Anxiety Inventory; STAI-S, Spielberger State Anxiety Inventory; BDI-II, Beck Depression Inventory–II).

Condition	Washing (*n* = 31)		Contamination (*n* = 31)		Control (*n* = 31)		*Comparison*
	μ	(*SD*)	μ	*(SD*)	μ	(*SD*)	*F*
Age	25.97	(8.88)	23.52	(3.39)	26.13	(9.98)	*F*_2,90_ = 1.05, *p* = 0.354
Education (years)	16.74	(3.65)	16.50	(2.68)	16.39	(2.69)	*F*_2,90_ < 1, NS
PI CF	19.10	(6.86)	19.35	(7.11)	20.19	(6.64)	*F*_2,90_ < 1, NS
OCI-R	20.55	(10.74)	20.10	(9.82)	24.48	(9.74)	*F*_2,90_ = 1.77, *p* = 0.177
Y-BOCS	3.76	(3.19)	3.23	(3.30)	3.94	(3.36)	^†^*F*_2,90_ < 1, NS
STAI-T	38.87	(8.81)	36.10	(9.66)	40.61	(10.53)	*F*_2,90_ = 1.71, *p* = 0.186
STAI-S	34.48	(8.73)	31.13	(6.75)	36.06	(10.62)	*F*_2,90_ = 2.52, *p* = 0.0864
BDI-II	6.97	(5.38)	8.10	(8.49)	7.16	(6.63)	^†^*F*_2,90_ < 1, NS
	***n***	**(%)**	***n***	**(%)**	***n***	**(%)**	
Sex (*n*/% female)	20	(64.5)	19	(61.3)	21	(67.7)	*Χ*^2^_2_ = 0.282, *p* = 0.869

^†^After applying a log_10_(*x* + 1) transformation, as for the main analysis (see text).

Additional data were missing on a small number of measures. Data for the number of sessions completed and for the circle-counting control task were lost for one (control) participant due to a technical problem. For one subject (in the washing condition), the test circles were not presented in a randomized fashion due to a technical error, but as this subject’s data did not deviate from that of other participants she was included. One subject’s post-intervention Y-BOCS data was lost and thus not analyzed. The final sample size for the Y-BOCS before/after analysis was thus 92 (washing condition *n* = 30, contamination *n* = 31 and control *n* = 31). One subject was excluded from the IED analyses as she only completed 2 stages (out of 9) on the task. The final sample size for the IED was therefore 92 (washing condition *n* = 31, contamination *n* = 31 and control *n* = 30).

### Smartphone intervention

All participants completed the 7-day intervention. Participants in all three conditions successfully completed the majority of smartphone sessions (*μ* = 24.98 out of a total of 28 sessions; *SD* = 2.84), and these did not differ by condition (whether analyzed untransformed or squared to reduce negative skew: *F*_2,89_ ≤ 1.29, *p* ≥ 0.28). Overall, participants appeared to watch the video footage on the application consistently. That is, there were very few inconsistencies between the number of circles shown on the videos and subsequently reported by participants (*μ* = 2.01 incorrect answers out of a total of 28, *SD* = 2.77), and these did not differ by condition (following a log_10_(*x* + 1) transformation, *F*_2,89_ = 1.25, *p* = 0.29).

### Baseline measures of contamination fears, OCD symptoms, and mood

Baseline performance on measures of contamination fears, OCD symptoms, and mood (PI CF, OCI-R, Y-BOCS, STAI-T, STAI-S, and BDI-II) did not differ by condition (Table 1).

### PI CF

**Figure 3:**
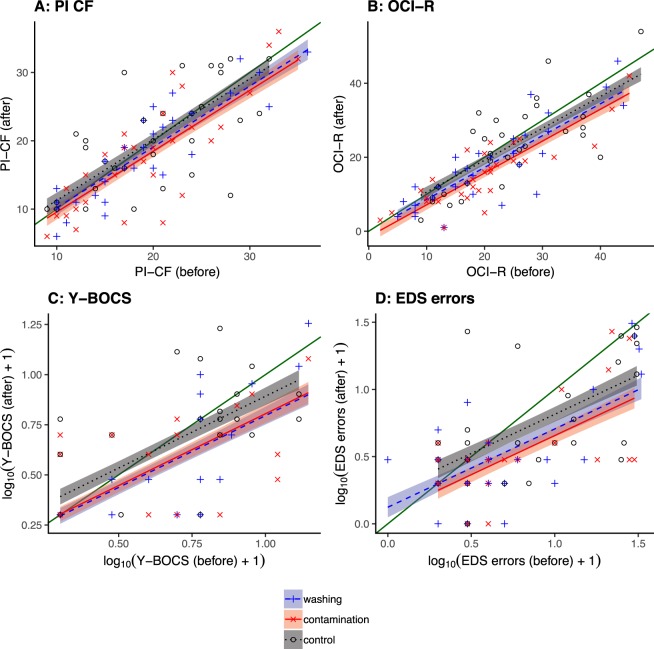
Scores before and after the smartphone interventions. (**A**) PI CF scores were not altered by the active interventions, compared to the control condition (see text). (**B**) OCI-R scores were reduced by both active interventions. (**C**) Y-BOCS scores were reduced by both active interventions. (**D**) EDS errors were reduced by both active interventions, compared to the control condition. Confidence ribbons indicate ±1 standard error. The green line with no confidence ribbon is the *x* = *y* line of “no change”; deviations from this in the control condition suggest e.g. practice effects, regression to the mean, or other nonspecific changes.

Neither intervention altered contamination fear scores (Fig. 3A). A single-slope ANCOVA model was used and residuals were normally distributed. There was no effect of treatment (*F*_*2*,*89*_ = 2.44, *p* = 0.0928).

### OCI-R

Both experimental interventions (i.e., the washing condition and contamination condition) reduced OCI-R scores (Fig. 3B). A single-slope ANCOVA model was found to be preferable; residuals were normally distributed. The effect of treatment was significant (*F*_2,89_ = 11.1, *p* = 5.15 × 10^−5^), with differences between the washing condition and control condition (*F*_1,59_ = 9.45, *p* = 0.0032), and between the contamination condition and the control condition (*F*_1,59_ = 19.3, *p* = 4.74 × 10^−5^), but no difference between the contamination condition and washing condition (*F*_1,59_ = 1.83, *p* = 0.181).

### Y-BOCS

Both interventions reduced Y-BOCS scores (Fig. 3C). Y-BOCS scores were subjected to a log_10_(*x* + 1) transformation to reduce skew and leptokurtosis; a single-slope ANCOVA model was found to be preferable. There was a main effect of treatment (*F*_2,88_ = 4.71, *p* = 0.0114). In pairwise ANCOVA comparisons, the washing condition differed from the control condition (*F*_1,58_ = 4.85, *p* = 0.0316) and the contamination condition differed from the control (*F*_1,59_ = 8.11, *p* = 0.00605), while the washing condition did not differ from the contamination condition (*F* < 1, NS).

### STAI-S

There was no effect of the intervention on STAI-S scores. STAI-S residuals showed only minor deviation from normality. A single-slope ANCOVA model was used; there was no effect of treatment (*F*_2,89_ < 1, NS).

### BDI-II

There was no effect of the intervention on BDI-II scores. BDI-II residuals were not normally distributed, with positive skew and leptokurtosis, but satisfied normality tests following a log_10_(*x* + 1) transformation; a single-slope ANCOVA model was preferred. There was no effect of treatment (*F*_2,89_ = 1.99, *p* = 0.143).

### Intradimensional–Extradimensional Set Shifting task

#### Extradimensional Shift Errors

As hypothesized, the two smartphone interventions (i.e., the washing and contamination condition) improved cognitive flexibility as assessed by a reduction in EDS errors (Fig. 3D), whereas no significant changes were observed in the control intervention. That is, both interventions (the washing and contamination condition) reduced EDS errors. A single-slope ANCOVA model was found to be preferable; residuals were normally distributed following a log_10_(*x* + 1) transformation. Ignoring information from baseline performance, the treatment effect appeared marginal (analysis of post-treatment EDS errors alone: *F*_2,89_ = 2.99, *p* = 0.0556). Taking into account baseline performance, the treatment effect became clear (effect of treatment: *F*_2,88_ = 4.95, *p* = 0.00918). Pairwise ANCOVAs showed that the washing condition reduced EDS errors compared to the control condition (*F*_1,58_ = 5.95, *p* = 0.0178), as did the contamination condition (*F*_1,58_ = 7.85, *p* = 0.0069). The two experimental interventions did not differ from each other (*F* < 1, NS).

#### Pre-extradimensional Shift Errors

As anticipated, neither intervention affected pre-EDS errors (i.e., errors in the stages before the extradimensional shift). Log-transformed pre-EDS errors deviated only very slightly from normality. Treatments had no effect on performance (*F*_2,88_ = 1.51, *p* = 0.226).

## Discussion

We present here two novel smartphone interventions found to improve cognitive flexibility and OCD symptoms in individuals with OCD-like contamination fears. It is striking that these changes in executive function and OCD symptomatology occurred after only one week of applying the intervention.

Improvements in cognitive flexibility, as assessed with the IED Set Shifting task, cannot be explained by practice effects, as they were not seen following the control intervention. These findings are especially intriguing as cognitive inflexibility (impaired set shifting) may represent the most prominent neuropsychological marker of OCD^13^, emphasizing the potential clinical utility of these smartphone interventions.

These findings are consistent with research showing that neuropsychological deficits in OCD are reversible: neural and metabolic dysfunction underlying such impairments can be improved using CBT^41^. Several studies found that behavioral therapy (administered over the course of weeks) ameliorated neuropsychological deficits, including set shifting, in patients with OCD^41–43^ (for a review see^44^).

In the current study, we found improvements on a task of cognitive flexibility in which poor performance is thought to reflect compulsive symptoms in OCD such as excessive washing rituals^13^. In addition, participants improved on a self-administered measure of distress associated with OCD symptoms (the OCI-R), and the Y-BOCS, which assesses OCD symptom severity. However, no changes were seen in self-reported scores of contamination fears. One possible explanation is that an intervention administered for a period of only one week (even in a clinical sample) would not directly affect self-perceived contamination fears. That is, this intervention might in many cases be too short to directly influence self-perceptions, especially if one identifies as averse to contamination (e.g., “I’ve always been a ‘germophobe’”). On the other hand, these data suggest that the interventions, albeit short, may improve underlying OCD-type tendencies and crucial cognitive processes like cognitive rigidity, perhaps outside one’s immediate awareness. Improvements in cognitive flexibility and OCD symptomatology, particularly in a clinical sample, might over time translate into detectable reductions in contamination fears.

As such, the smartphone interventions may have improved cognitive flexibility and OCD symptoms by influencing compulsive-like behaviors and propensities. The vicarious relief intervention (the washing condition) may have provided acute “doses” of relief, such that any washing urges and perhaps subsequent compulsive-like behaviors were either eliminated or reduced after using the application. This might have led to a reduction in conditioned fear associated with refraining from performing the compulsive behavior. Similarly, the vicarious desensitization intervention (the contamination condition) may have provoked disgust-related anxiety that diminished over the course of the treatment. Such repeated and systematic exposure could have caused participants to become increasingly desensitized to real-life stimuli that would normally trigger contamination concerns (e.g., when shaking hands), and in turn compulsive-like behaviors.

Anxiety is a crucial component of the cognitive architecture of OCD^45^. It is believed to bias cognitive systems towards habitual and rigid thinking, leading to impairments in attentional control, including inhibition and shifting^46^. According to one hypothesis, over-reliance on “habit systems” underlies symptoms of compulsivity in OCD^47^. In the present study, the interventions ostensibly did not reduce overall state anxiety levels and mood. Instead, the data indicate that the interventions had a more direct and specific effect on OCD-like tendencies (and perhaps anxiety and stress specific to such propensities). This in turn might have helped participants employ more effective cognitive strategies, and possibly rely to a greater extent on goal-directed cognitive systems, thus allowing them to think in a more flexible (less rigid) manner.

Consistent with this account, several participants in the washing condition reported (prior to the debriefing at the end of the second session) that the intervention made them feel relaxed and had a soothing effect. As one participant noted, “[it felt as if] I had washed my hands, so I didn’t need to wash my hands anymore… my hands were clean after using the app”. Another participant reported, “I was surprised that watching myself washing hands produces relief”; and another that “if I am commuting, [e.g.,] on the bus and touch something contaminated and can’t wash my hands for the next two hours, the app would be a sufficient substitute”. Likewise, participants in the contamination condition remarked that they initially felt disgusted when watching the video footage, but that such feelings were reduced over time. One participant added, “the first half of the week, I found the video disgusting. Second half, not as disgusting…”; and another, “in real life one would not touch something as disgusting… touching something so disgusting becomes normalized” (by watching the video). One participant noted, “my contamination and washing tendencies reduced a lot. For example, if I put the rubbish out and touch the bin, I would normally wash my hands immediately. But after I started to use the app, I felt like it would be silly to wash my hands…” and “I have become desensitized to the video and other things as well. If I normally were to wipe a kitchen worktop, I would throw the cloth away because I felt it was disgusting to clean that cloth for another time. But since using the app I now use the cloth, clean it, and use it again another time”; thus, “…it generalized to other things, so I felt like other things weren’t as disgusting as I previously thought they were.”

These reports, while anecdotal, provide valuable insights about participants’ subjective psychological state while exposed to the smartphone interventions. They also dovetail nicely with research demonstrating that disgust, and relief from disgust, can be induced via a proxy stimulus (i.e., vicariously) in individuals with OCD symptoms^9^.

Viewing oneself (versus another person) on film might be advantageous for several reasons. Self-identification with the agent performing the relief- or contamination-inducing behavior (washing hands or being contaminated) might enhance any empathetic response. Also, merely the memory of oneself performing such a salient behavior (i.e., one that eliminates or provokes contamination obsessions) is likely to help trigger an emotional reaction^48^. Moreover, compulsions in OCD, such as excessive hand-cleansing rituals, can be highly idiosyncratic, visibly differing from one person to the next^49^. This reality was echoed in our recent study^9^: to maximize vicarious relief sensations felt by watching someone else washing their hands, participants would sometimes specify how the other person’s cleansing ritual should be performed. Displaying video footage of participants performing their own handwashing therefore potentially ensures that this ritual is sufficiently personalized to maximize relief.

Central limitations of traditional therapies for OCD, such as ERP, include cost, inconvenience of delivery (e.g., participant travel), and intolerability of the treatment procedures^5^, resulting in considerable dropout rates. In the present study, participants showed high levels of adherence to the smartphone interventions: all participants completed the entire one-week intervention; and although participants had to complete as many as four sessions a day within fixed time periods, very few sessions were missed. Likewise, participants were generally attentive to the video footage on the application. These findings demonstrate the practical utility of the interventions and suggest that they could potentially overcome some of the challenges associated with traditional OCD therapies.

Future research, conducted in large clinical groups with OCD, should explore further the efficacy and feasibility of these novel treatments. Imaging methods would be useful to map improvements in cognitive function and OCD symptomatology onto neural correlates. Such research should also directly compare the two interventions. It is conceivable the effectiveness of each intervention depends on the severity and number of months since onset of symptoms.

In summary, we introduce two smartphone interventions and show that they improve cognitive function and OCD symptoms after only one week in individuals with contamination fears. These interventions could potentially have significant public health and societal impact. They are forms of “technology-based personalized medicine” that are not only inexpensive and accessible but can be tailored for individual patients. They also have the potential for widespread implementation and could potentially reach communities that do not have access to adequate mental health care.

## Data Availability

The raw data are available from the corresponding author upon request.
